# Supplementary material for: SMORES: a simple microfluidic operating room for the examination and surgery of Stentor coeruleus
Source: Sci Rep. 2024 Apr 15;14:8684. doi: 10.1038/s41598-024-59286-y (PMC11018760; doi:10.1038/s41598-024-59286-y)
Supplement: Supplementary file 1 — Supplementary Information 1. [file 41598_2024_59286_MOESM1_ESM.pdf]

Supplementary Information

SMORES: A Simple Microfluidic Operating Room for the Examination and Surgery of *Stentor coeruleus*

Kevin S. Zhang, Ramon Rodriguez, and Sindy K. Y. Tang*
*Corresponding author, sindy@stanford.edu

Contents:

Supplementary Figure S1. Additional details on the design of SMORES.
Supplementary Figure S2. Image processing pipeline.

See online version for:

Supplementary Video S1. Compressed *Stentor* cell inside a cage trap.
Supplementary Video S2. Uncompressed *Stentor* cell inside a cage trap.
Supplementary Video S3. Confocal z-stack of a *Stentor* cell.
Supplementary Video S4. Retrieval of a laser ablation-wounded cell from a microcompressor.

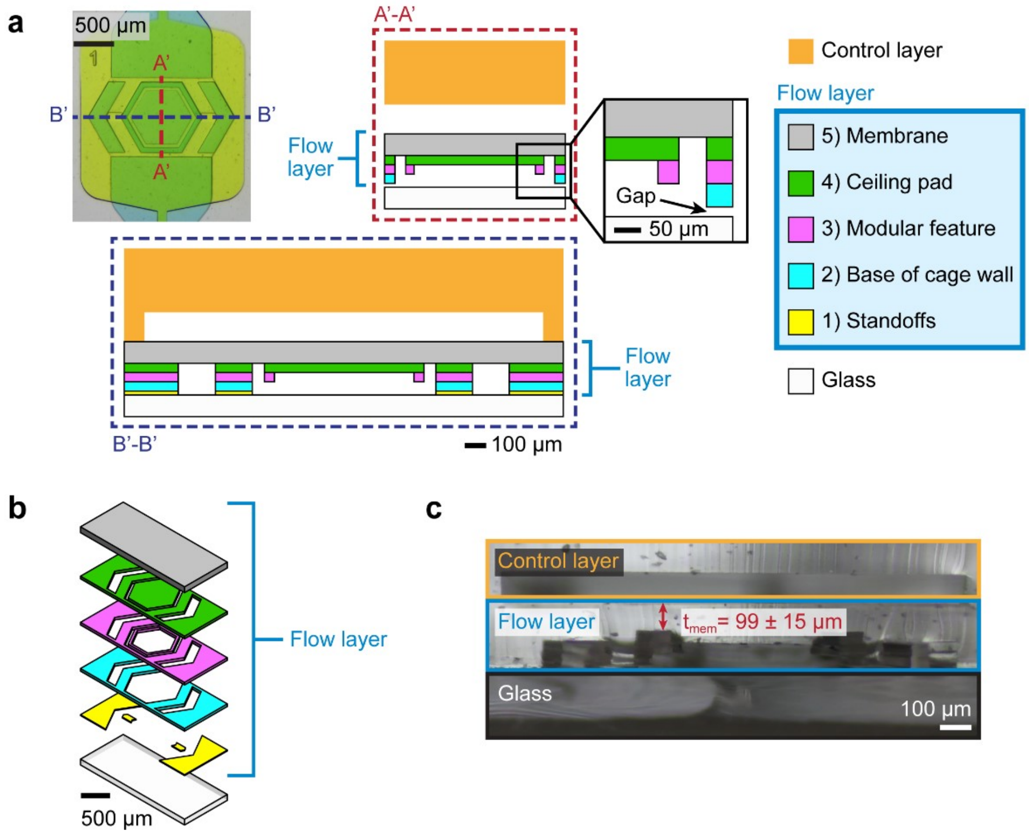

Supplementary Figure S1. Additional details on the design of SMORES. **(a)** Cross-sectional views of the SMORES platform showing the layered components comprising the cage trap in the flow layer. **(b)** Exploded view of the layered components comprising the cage trap in the flow layer. **(c)** Cross-section view of a SMORES device that has been cut open approximately along the B'-B' section line. Membrane thickness t_{mem} reported as mean ± standard deviation (n=15 devices). Note some distortion of the PDMS features occurs due to the cutting.

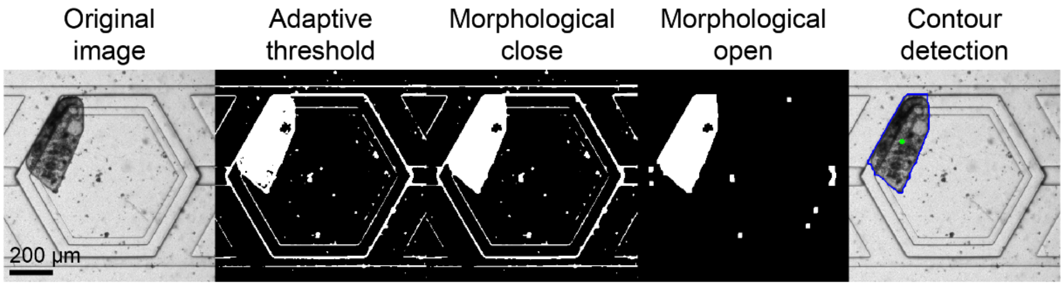

Supplementary Figure S2. Image processing pipeline. An adaptive threshold identifies dark regions in the original image. A morphological close reduces the noise present inside the cell body. A morphological open removes the thin borders of the cage trap features. Finally, contour detection locates the centroid of the cell. Note the image processing occasionally introduces artefacts into the detected cell contour; however, these artefacts do not change the results (also see Supplementary Videos S1 and S2).
